# Color-Transfer-Enhanced Data Construction and Validation for Deep Learning-Based Upper Gastrointestinal Landmark Classification in Wireless Capsule Endoscopy

**DOI:** 10.3390/diagnostics14060591

**Published:** 2024-03-11

**Authors:** Hyeon-Seo Kim, Byungwoo Cho, Jong-Oh Park, Byungjeon Kang

**Affiliations:** 1 Graduate School of Data Science, Chonnam National University, Gwangju 61186, Republic of Korea; kimhs9574@gmail.com; 2 Korea Institute of Medical Microrobotics, Gwangju 61011, Republic of Korea; bwcho@kimiro.re.kr (B.C.); jop@kimiro.re.kr (J.-O.P.); 3 Department of Artificial Intelligence Convergence, Chonnam National University, Gwangju 61186, Republic of Korea

**Keywords:** wireless capsule endoscopy, deep learning, landmark classification

## Abstract

While the adoption of wireless capsule endoscopy (WCE) has been steadily increasing, its primary application remains limited to observing the small intestine, with relatively less application in the upper gastrointestinal tract. However, there is a growing anticipation that advancements in capsule endoscopy technology will lead to a significant increase in its application in upper gastrointestinal examinations. This study addresses the underexplored domain of landmark identification within the upper gastrointestinal tract using WCE, acknowledging the limited research and public datasets available in this emerging field. To contribute to the future development of WCE for gastroscopy, a novel approach is proposed. Utilizing color transfer techniques, a simulated WCE dataset tailored for the upper gastrointestinal tract is created. Using Euclidean distance measurements, the similarity between this color-transferred dataset and authentic WCE images is verified. Pioneering the exploration of anatomical landmark classification with WCE data, this study integrates similarity evaluation with image preprocessing and deep learning techniques, specifically employing the DenseNet169 model. As a result, utilizing the color-transferred dataset achieves an anatomical landmark classification accuracy exceeding 90% in the upper gastrointestinal tract. Furthermore, the application of sharpen and detail filters demonstrates an increase in classification accuracy from 91.32% to 94.06%.

## 1. Introduction

**Figure 1:**
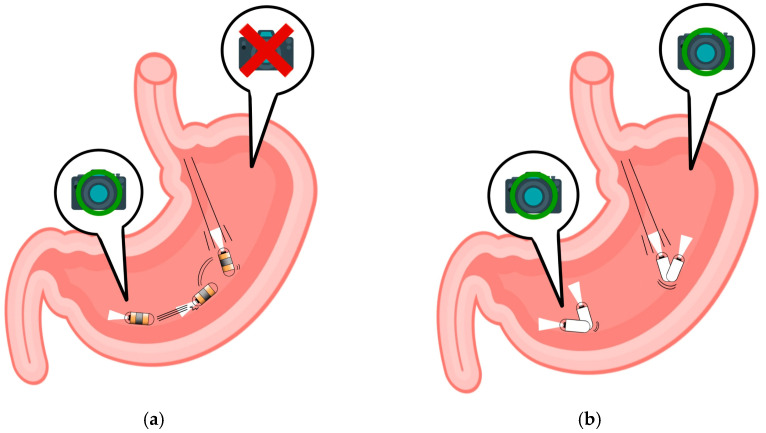
Evolving capsule endoscopy technologies: (**a**) wireless capsule endoscopes; (**b**) MCCE.

The global endoscopy market is experiencing steady increase, transcending geographical boundaries, as highlighted by its expansion beyond the confines of the United States and China [1]. Conventional wired endoscopy, while a cornerstone in diagnostics, has several limitations, such as the need for sedation, active involvement, and specialized techniques [2]. In contrast, wireless capsule endoscopy (WCE) offers a more patient-friendly alternative, with individuals simply ingesting a capsule with water. The transmitted capsule images are subsequently reviewed by endoscopists, typically providing diagnostic results within an average of 30–40 min [3]. The simplicity and convenience of wireless capsule endoscopy have resulted in a recent surge in popularity, showing the potential to outpace wired endoscopy [4]. However, the current focus of WCE is predominantly on the small intestine [5]. This preference arises from the narrow luminal diameter of the small intestine within the gastrointestinal tract, enabling WCE to effectively capture essential imaging areas [5,6]. Nonetheless, technological advancements are propelling efforts to expand WCE capabilities for observing the upper gastrointestinal tract [7,8,9,10]. Notably, magnetic-controlled capsule endoscopy (MCCE) has emerged as a promising alternative to passive and uncontrollable WCE, demonstrating the capability to image landmark areas in the upper gastrointestinal tract [11]. Figure 1 presents a comparative concept between the existing WCE and the ongoing development of MCCE.

Despite the increasing interest in upper gastrointestinal WCE, there is a limited body of research dedicated to identifying anatomical landmarks through WCE imaging [12]. This limitation is attributed to the nascent nature of this research domain [13,14]. Xu et al. proposed a multitask anatomy detection network for gastroscopy, successfully classifying 10 upper gastrointestinal anatomical structures with an average detection accuracy of 93.74% and classification accuracy of 98.77% [15]. Takiyama et al. conducted an automatic anatomical classification study on 27,335 images categorized into esophagogastroduodenoscopy (EGD)-based larynx, esophagus, stomach, and duodenum using a GoogleNet-based architecture [16]. They further extended their study to classify and evaluate upper, middle, and lower stomachs using a validation set of 13,048 items, achieving a four-class classification accuracy of 97% and a three-position study accuracy of 99%. As indicated above, while studies exist on the identification of upper gastrointestinal landmarks through wired conventional endoscopy, research on upper gastrointestinal landmarks with WCE images has only recently emerged [17,18]. Further investigations are needed for landmark identification in this specific context. The primary objective of this study was to propose a method for providing uniform and high-accuracy imaging with capsule endoscopic images, incorporating a prelandmark for the upper gastrointestinal tract. This approach aims to facilitate the imaging identification of active capsules within the upper gastric tract, anticipating their future commercial availability. Historically, capsule endoscopic images required video processing due to their lower quality and darker nature than wireless endoscope images. In our effort to enhance WCE images for improved accuracy, akin to wired endoscope images, we engaged in image preprocessing. Consequently, WCE images for the upper gastrointestinal tract became essential for achieving this purpose. However, a significant challenge in this field is the absence of a dedicated public dataset for the WCE images of upper gastrointestinal landmarks, excluding the small and large intestines [19]. Existing public datasets primarily focus on classifying normal and abnormal diseases across unspecified segments of the gastrointestinal tract [20]. While capsule endoscopy images of the stomach are available, they often lack coverage of essential imaging areas based on EGD, making it difficult to identify landmarks.

To address this gap, a realistic WCE dataset was created to contribute to the advancement of future upper gastrointestinal WCE. To validate the similarity of this dataset to actual WCE images, a small intestine dataset that includes both wired endoscopy and WCE endoscopy datasets was used, namely the Kvasir and Kvasir-capsule datasets, containing landmark images of the normal small intestine [19,21]. Our hypothesis posited that applying the colors of the Kvasir-capsule to the Kvasir datasets using color transfer techniques would result in similar accuracy after training with DenseNet169. Testing this hypothesis affirmed that landmark images subjected to color transfer closely resemble actual WCE images. Subsequently, thresholds and error ranges were established based on this comparison, which were then applied to the upper gastrointestinal landmark dataset to create a comprehensive dataset [22,23]. The WCE image dataset for the upper gastrointestinal tract, validated through this hypothesis, demonstrates performance similar to that obtained using WCE images captured from the actual upper gastrointestinal tract. Moreover, this dataset has the potential to enhance the accuracy of identifying landmarks when applied to wired endoscopy images, following image preprocessing. Our intended preprocessing includes utilizing a dataset with implemented color transfer and leveraging deep learning techniques to improve the accuracy of classifying images depicting upper gastrointestinal anatomical landmarks. After this process, we anticipate that even low-quality WCE images will demonstrate uniform and high accuracy comparable to high-quality wired endoscopes. Moreover, the dataset is expected to successfully be used to identify the landmarks required for EGD-based imaging. This is anticipated to contribute to the development of WCE for the upper gastrointestinal tract, slated for future commercialization.

This paper is organized as follows: Section 2 provides an overview of upper gastrointestinal tract landmark classification, introduces the dataset, and outlines experiments for model selection and resolution comparison. It details the hypothesis formulation, the method for selecting an image similarity proof method to create a dataset mimicking a WCE image, the image similarity proof experiment using the Kvasir and Kvasir-capsule datasets, the production of a WCE dataset for the upper gastrointestinal tract, and the image similarity verification process, including the resulting threshold and error range selection. In Section 3 (Results), we describe an experiment confirming landmark classification accuracy using the DenseNet169 model with the WCE datasets from Section 2. The section explains the results of enhanced classification accuracy achieved through image preprocessing. Section 4 (Discussion) discusses the experimental results, considers limitations, and proposes future research directions. Section 5 (Conclusions) provides a summary of this research.

## 2. Materials and Methods

### 2.1. Related Research and Model Selection

The adoption of deep learning technology has significantly impacted the field of medical imaging, and this influence has been further amplified through the use of CNNs [24,25]. In particular, various deep learning models based on CNNs have been developed, presenting a novel approach to medical image analysis. Within this domain, research utilizing CNNs has flourished, resulting in the creation of numerous deep learning models tailored for CNNs [26,27,28]. Previously, there was heavy reliance on the performance of capsule endoscopy equipment, but recent developments in deep learning technology have spurred a range of CNN-based innovative research. As a related research case, we analyzed research progress by year through the examination of anatomical landmark data captured via a wireless capsule endoscope. Table 1 illustrates the research progress over the years based on anatomical landmark data collected using wireless capsule endoscopes [14,29,30,31,32,33,34,35,36,37].

Research to date has used a variety of CNN models. However, several studies have shown wide and inconsistent limits in accuracy [14,30,31,32,33,34,35,36]. The performance of these models can vary depending on the characteristics of each dataset. In the pursuit of an effective CNN model for our dataset, we applied deep learning to the original dataset using commonly utilized models: ResNet, DenseNet, and Inception, as indicated in the latest studies presented in Table 1. Among these models, the DenseNet model exhibited the highest average classification accuracy of 93.28%. Given the various versions of the DenseNet model, including DenseNet121, DenseNet169, and DenseNet201, we systematically trained and evaluated these models to identify the optimal performer. The deep learning accuracy results for each model on the original data were 92.26% for the DenseNet121 model, 93.28% for the DenseNet169 model, and 91.17% for the DenseNet201 model. Ultimately, the DenseNet169 model was selected for our study due to its highest classification accuracy. DenseNet is a model specialized for transfer learning and excels in classification problems using small datasets, making it well suited for our research [38,39]. Table 2 presents the deep learning results for each model on the original dataset. Notably, DenseNet169 has exhibited superior performance in various contexts, as reported in other studies [40]. Additionally, Abbas et al. successfully employed DenseNet169 in their research on a five-stage automatic detection and classification system for hypertensive retinopathy, demonstrating its efficacy in classification tasks [41]. In a study by Farag et al., a novel architecture based on DenseNet169 was created to classify the severity of diabetic retinopathy, achieving an impressive 97% accuracy in severity ratings [42]. Based on this collective evidence, the DenseNet169 model was chosen for this study due to its anticipated high performance in classification accuracy.

### 2.2. Original Dataset

**Figure 2:**
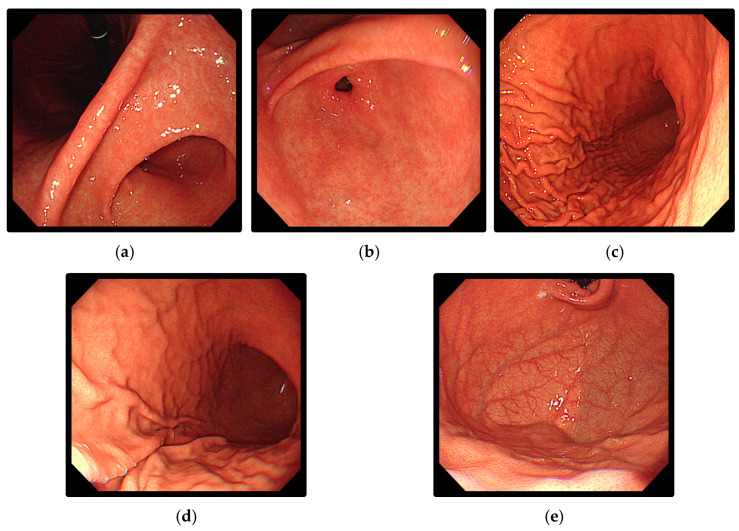
Examples of upper gastrointestinal landmarks: (**a**) Angulus; (**b**) Antrum; (**c**) Body A; (**d**) Body B; (**e**) Cardia and Fundus.

In this study, a dataset comprising 2526 images capturing upper gastrointestinal landmarks obtained from endoscopic procedures conducted at Yonsei Severance Hospital was utilized. The dataset included images of five distinct landmarks: Angulus, Antrum, Body A, Body B, as well as Cardia and Fundus. Table 3 provides the distribution of images in the original dataset, and Figure 2 illustrates an exemplary image from the dataset, showcasing instances of the five classes of upper gastrointestinal tract landmarks.

### 2.3. Hypothesis Formulation

**Figure 3:**
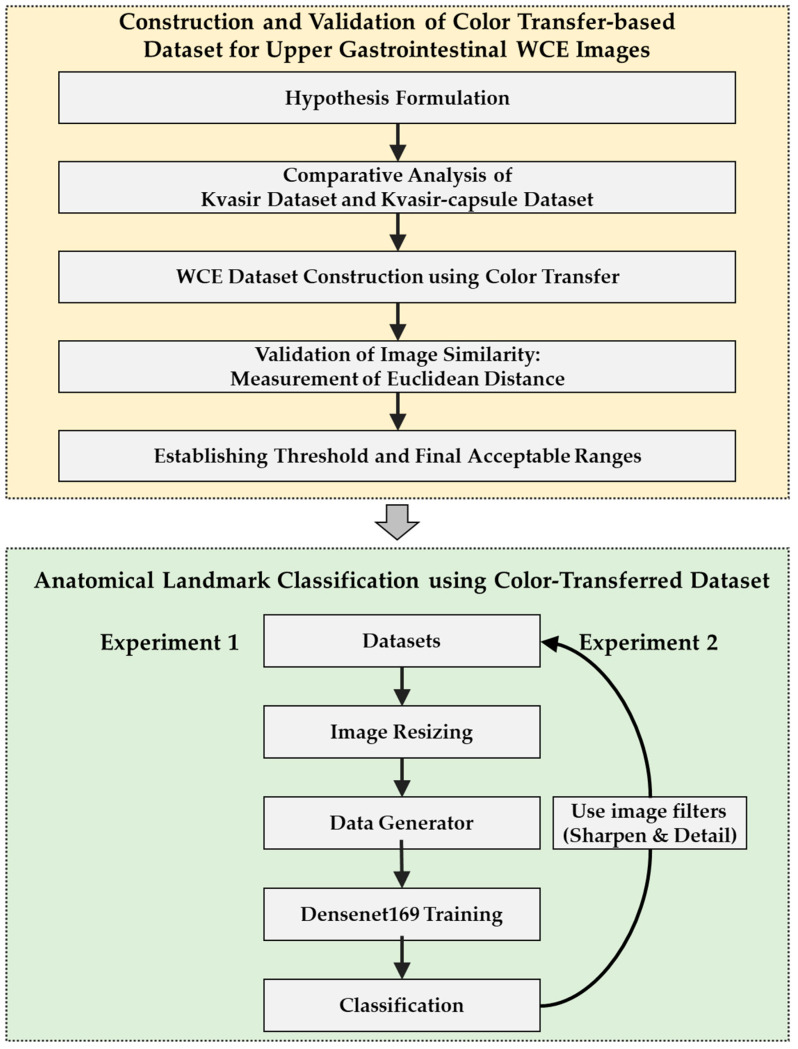
Workflow of the study.

Figure 3 depicts the workflow of the flow study chart, detailing the progression. Initially faced with challenges in obtaining wireless capsule endoscopy (WCE) images targeting upper gastrointestinal landmarks, a hypothesis was developed to create an alternative dataset. Our hypothesis posited that endoscopic images of similar quality to WCE images would yield comparable outcomes when applied in practice. To validate this hypothesis, the Kvasir-capsule dataset, which contains WCE images, and the Kvasir dataset, which comprises wired endoscopy images, were used. Due to the limited availability of WCE images, especially for the small intestine, comparative analysis of the two datasets was deemed suitable. It was anticipated that applying color transfer to the Kvasir dataset to mimic the WCE image colors of the actual small intestine would yield results similar to those of the Kvasir-capsule dataset. To validate this expectation, a validation plan was applied to postulate that if color-transfer-induced similarity was confirmed through these results, analogous outcomes could be expected for upper gastrointestinal landmarks. Assessment of this similarity involved quantifying image comparison using the Euclidean distance.

### 2.4. Validation of Alternative WCE Datasets

#### 2.4.1. Selection of the Image Similarity Measurement Method

To illustrate the similarity between color-transferred wired endoscope images and actual WCE images, this study’s approach involved assessing image similarity. Several methods exist for evaluating similarity between images, with the Euclidean distance (a distance-based measurement) and cosine similarity (an angle-based measurement) being among the most commonly employed [43,44,45]. The Euclidean distance quantifies the distance between vectors and gauges their similarity, with a closer proximity to 0 indicating greater similarity [46]. Heidari et al. utilized color-based descriptors as image features and applied Euclidean distance for image comparisons [47]. Furthermore, Wang et al. introduced an intuitive Euclidean distance measure for images, referred to as image Euclidean distance [48]. The formula for Euclidean distance is expressed as follows:

(1) $$||p-q||=\sqrt{\left(p-q)\cdot (p-q\right)}=\sqrt{{\left||p|\right|}^{2}+{\left||q|\right|}^{2}-2p\cdot q}$$

here, p and q are vectors in multidimensional space.

Conversely, cosine similarity calculates image similarity by considering the cosine angle between vectors, yielding values between −1 and 1 [49]. The formula for cosine similarity is given by

(2) $$similarity=\cos\left(\theta \right)=\frac{A\cdot B}{\left||A||+||B|\right|}=\frac{\sum_{i=1}^{n}A_{i}\times B_{i}}{\sqrt{\sum_{i=1}^{n}{\left(A_{i}\right)}^{2}}\times \sqrt{\sum_{i=1}^{n}{\left(B_{i}\right)}^{2}}}$$

Here, ||A|| and ||B|| represent the Euclidean norms of vectors A and B, respectively.

Given the focus on determining color similarity rather than shape, the Euclidean distance suitable for this study’s objectives was discovered, especially when shape value deviations were minimal. In cases where the deviation of each shape value was substantial, cosine similarity proved effective [50]. Considering the difficulty in extracting features from the upper gastrointestinal landmarks compared with from other datasets, this study placed less emphasis on shape values. Consequently, the Euclidean distance was determined to be more aligned with this study’s objectives. However, during the measurements of Euclidean distance, subtle differences in shape across each class were maintained [51]. The VGG16 model, pre-trained on the ImageNet dataset, was employed for Euclidean distance measurements [52].

#### 2.4.2. Comparative Analysis of the Kvasir and Kvasir-Capsule Datasets

To validate our hypothesis, two distinct proof procedures were implemented. First, a color transfer from the Kvasir-capsule image to the Kvasir image was executed. Utilizing the well-known color transfer algorithm, as reported by Reinhard et al., this algorithm transmits only red, green, and blue (RGB) colors by calculating the mean and standard deviation (SD) of the RGB channel of the target image in the original image [53]. Notably, the original image’s features remain untouched, and only the color is transferred to the resulting image. In a similar context, Yin et al. conducted a study leveraging color transfer to identify faces by race through the transfer of skin color [54]. The formula for color transfer is expressed as follows:

(3) $$I_{k}=\frac{\sigma_{t}^{k}}{\sigma_{A}^{k}}\left(S^{k}-mean\left(S^{k}\right)\right)+mean\left(T^{k}\right)\;,\;k=(l,\alpha ,\beta )$$

here, $I_{k}$ represents the color-transferred image for a specific channel k. $\sigma_{t}^{k}$, $\sigma_{A}^{k}$, $S^{k}$, and $T^{k}$ denotes SD of the target image channel k, SD of the source image channel k, the source image channel k, and the target image channel k, respectively. k=(l,α,β) denotes the specific color channel, where l represents an achromatic channel; and α and β represent chromatic yellow-blue and red-green opponent channels, respectively.

**Figure 4:**
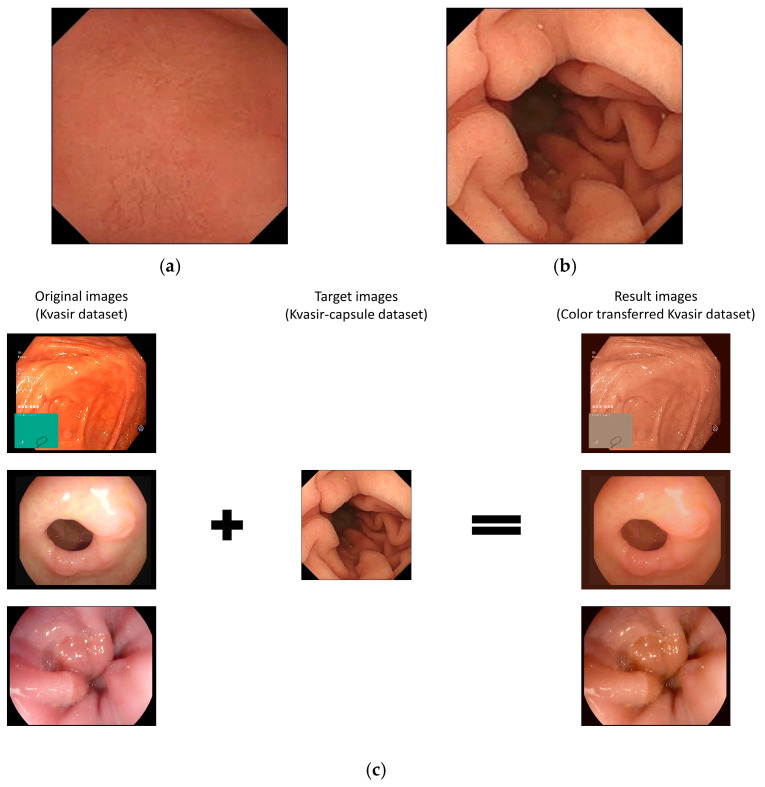
Illustration of Color Transfer and Kvasir-capsule Images: (**a**) Example image of Kvasir-capsule (1); (**b**) Example image of Kvasir-capsule (2). These two images serve as examples for calculating Euclidean distance. (**c**) Demonstration of the color transfer from the Kvasir-capsule image to the Kvasir dataset image. The images are arranged in the order of normal cecum, normal pylorus, and normal z-line areas from the top.

Subsequently, the color-transferred Kvasir and Kvasir-capsule datasets were trained to classify classes using DenseNet169 with a 320 × 320 input size. The classification accuracy for the color-transferred Kvasir dataset reached 98.95%, whereas the Kvasir-capsule dataset achieved an accuracy of 95.92%. Although the Kvasir dataset with color transfer exhibited a slightly higher error of approximately 3%, both datasets demonstrated an accuracy exceeding 95%, indicating similar performance. Further analysis using Euclidean distance to measure image similarity yielded values ranging from approximately 0.8 to 1.2. Table 4 provides a breakdown of Euclidean distance values for three classes, each consisting of 500 images from the Kvasir dataset with color transfer based on the Kvasir-capsule dataset. These two validation approaches collectively confirmed that the color-transferred WCE image exhibited deep learning accuracy akin to that of the actual WCE image. Figure 4 shows the illustration of color transfer and Kvasir-capsule images.

#### 2.4.3. WCE Dataset Construction Using Color Transfer

Building on the comparison of the results between the color-transferred Kvasir and Kvasir-capsule datasets, a WCE dataset for the upper gastrointestinal tract was created by applying color transfer from capsule endoscopy to images obtained through traditional wired endoscopy. To address the initial imbalance in the number of images across classes, data augmentation techniques were employed, including top-down inversion, left-right inversion, and a 20-degree tilt, as detailed in Table 3. This augmentation process resulted in 6400 photos, with 1280 photos in each class [55].

**Figure 5:**
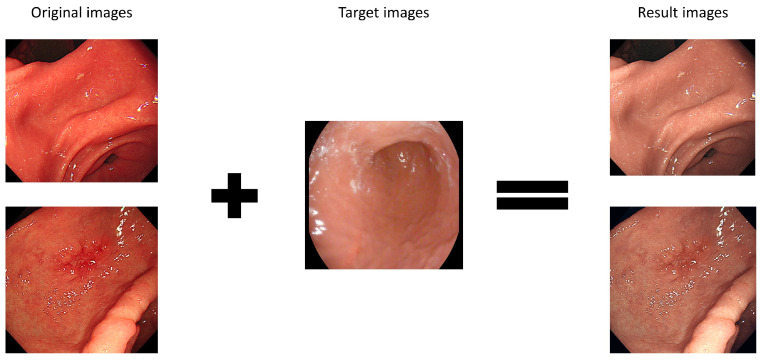
Example images demonstrating color transfer. This process illustrates the application of color transfer to the original images based on the target images. The original images were sourced from wired endoscopy, and the target images represent examples from one of the three target datasets, namely, the Gastrointestinal Bleeding WCE dataset.

**Figure 6:**
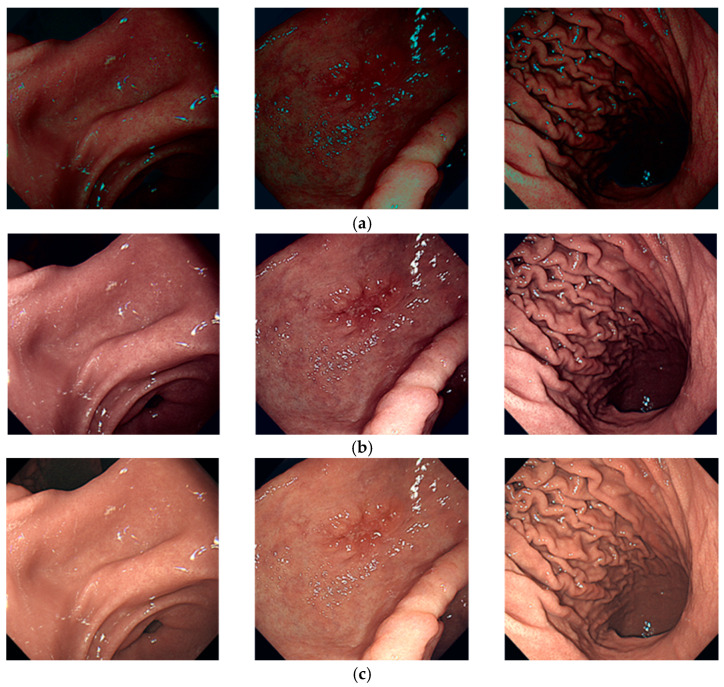
Color transferred-dataset from the three target images: (**a**) L dataset; (**b**) R dataset; (**c**) G dataset. Each panel displays representative examples from their respective datasets. The variations in image colors are attributed to differences in WCE equipment and the shooting environments of target images.

Using datasets from Liao et al. [56], Rahman et al. [57], and the Gastrointestinal Bleeding WCE dataset [58], these three datasets were selected as target images. The color information extracted from these target datasets was subsequently applied to the original images obtained through wired endoscopy at Severance Hospital. Consequently, a new dataset of WCE images for the upper gastrointestinal tract was obtained. Figure 5 illustrates example images demonstrating color transfer, and Figure 6 displays the resulting color-transferred images based on the three target datasets. The resulting image datasets were designated and produced through the application of color transfer as the L, R, and G datasets, respectively. The L dataset was derived from Liao et al. [56], the R dataset from Rahman et al. [57], and the G dataset by focusing on normal images within the Gastrointestinal Bleeding WCE dataset [58]. These specific datasets served as references for our study.

#### 2.4.4. Validation of Image Similarity: Measurement of Euclidean Distance

Image similarity was assessed by comparing three reference images with actual upper gastrointestinal tract WCE images using the three resultant datasets. The Euclidean distance was calculated for each of the five classes within the three datasets. Among the distance values derived from 1280 images for each class, the analysis focused on identifying the closest and farthest values from the target image. The results of the Euclidean distance measurements for the target image and the three datasets are presented in Table 5, with 1280 images used for each class. The left side of the table denotes the closest distance, and the right side represents the farthest distance.

#### 2.4.5. Establishing Threshold and Final Acceptable Ranges

The average similarity values obtained using the Euclidean distance measure ranged between 0.8 and 1.2. To define the threshold, the method outlined by Tian et al. was adopted [23]. As Tian et al. lacked the necessary error data for measuring outliers, a simulation was conducted, and a threshold was established. Consequently, the distance value threshold was set at an average value of 1.0, with an error range of within +0.2. The value of −0.2 was excluded, as a proximity to 0 in Euclidean distance indicates higher similarity. Therefore, the final acceptable range was set within Euclidean distances of up to 1.2.

### 2.5. Experimental Methods

An experiment was designed to classify upper gastrointestinal landmarks using deep learning based on three datasets. The training, validation, and test datasets comprised 3840, 1280, and 1280 pieces of data, respectively, with a distribution ratio of 6:2:2. The chosen model for the experiment was DenseNet169, which was implemented using TensorFlow, with a batch size of 16, and stochastic gradient descent as the optimizer. Two experiments were conducted within this experimental framework:

- Accuracy comparison across three datasets based on five image sizes;
- Accuracy evaluation for datasets with five image sizes and different image preprocessing techniques (sharpen and detail filters).

The first experiment involved the classification of upper gastrointestinal landmarks using DenseNet169 with various image sizes. Although the prevalent size for commercial WCE is 320 × 320 [59], in previous research, Iqbal et al. and Handa et al. used a size of 128 × 128 in capsule endoscopy [60,61], which was undertaken to enhance accuracy and speed. The analysis covered input images of sizes 128 × 128, 256 × 256, 384 × 384, and 512 × 512. The goal was to align the results with those of previous studies and consider the image quality of current commercialized WCE images [62,63]. The second experiment aimed to evaluate the accuracy of the datasets with five image sizes by incorporating distinct image preprocessing techniques such as the sharpen and detail filters. These experiments collectively explored the impact of various image sizes and preprocessing techniques on the classification of upper gastrointestinal landmarks using deep learning with DenseNet169.

## 3. Results

### 3.1. Accuracy Comparison Experiment of L, R, and G Datasets

#### 3.1.1. Accuracy Comparison among Three Datasets

This study compared the landmark classification accuracy of three datasets based on DenseNet169. In the experiment, on the L dataset, an average classification accuracy of 92.62% was achieved; on the R dataset, the average accuracy was 92.41%; and on the G dataset, the average accuracy was 92.38%. Notably, the highest classification accuracy was achieved on average for the L dataset. For all three datasets, an average classification accuracy of 92% was maintained.

#### 3.1.2. Classification Accuracy for Different Input Image Sizes

Among the five image sizes, the highest landmark classification accuracy was achieved for 128 × 128 and 256 × 256. Upon analysis, two assumptions were made. First, DenseNet uses a dense block composed of several convolutional layers, and the dense connection between these layers facilitates information reuse. The experiment suggested that smaller images resulted in more accurate landmark classifications than larger images, contributing to increased accuracy. Second, the relatively small size of the dataset (around 6400 images) allowed for effective transfer learning with small data, implying that larger input images may result in lower accuracy. Table 6 confirms that based on these factors, the accuracy experiences a slight decrease as the image size increases.

#### 3.1.3. Classification Accuracy among Different Classes

**Figure 7:**
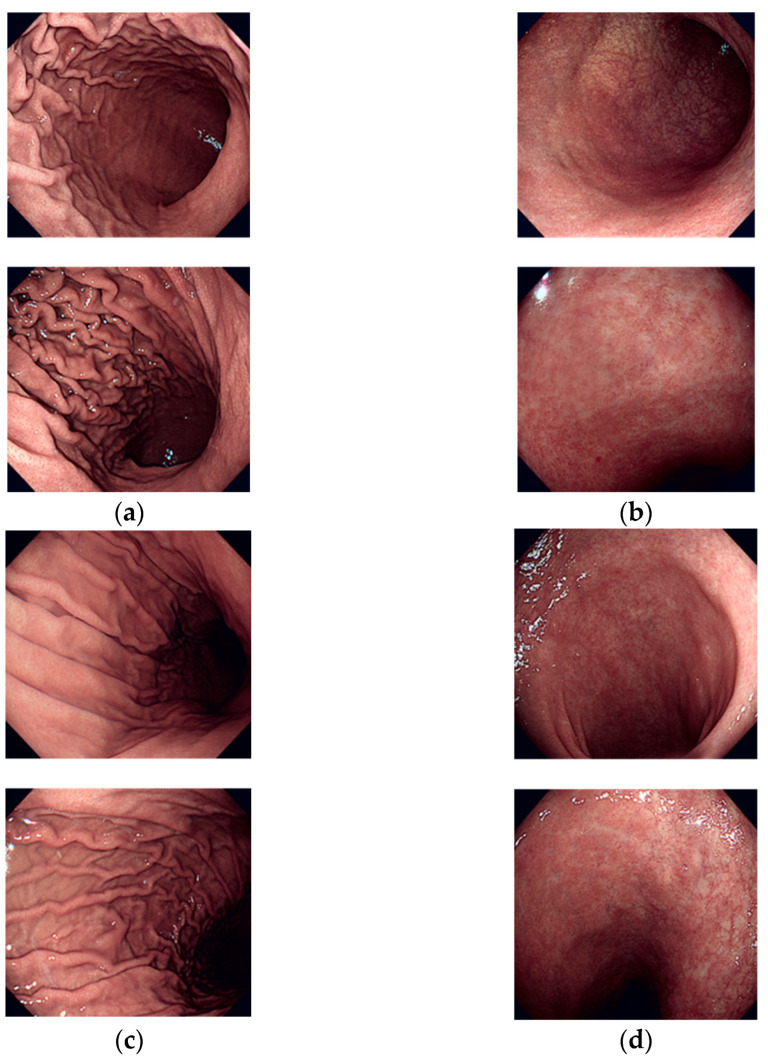
Examples of correctly and incorrectly classified images for the Body A and Body B classes: (**a**) correctly classified images of Body A; (**b**) incorrectly classified images of Body A; (**c**) correctly classified images of Body B; (**d**) incorrectly classified images of Body B.

In the experiment, the highest accuracy was achieved for the Cardia and Fundus classes among the five classes, whereas the lowest accuracy was achieved for the Body A class. Upon analyzing the dataset images, the distinct narrow features of the esophagus were prominent, especially around the Cardia portion in the Cardia and Fundus class. The Fundus class was characterized by several parallel upper wrinkles. These unique feature points differed from those in other classes, suggesting successful learning in extracting these distinctive features through deep learning. Consequently, probability of misclassification for images in the Cardia and Fundus class was lower. Conversely, the lowest accuracy was achieved for Body A. Despite representing the same body part as the Body B class, Body A was captured in the greater curvature area, whereas Body B was photographed in the lesser curvature area, resulting in different shooting positions. However, the differences in wrinkle shapes between the two classes were not significant, and numerous images featured smooth stomach walls, posing challenges in extracting distinctive feature points for both classes. Consequently, a high probability of misclassification arose due to the difficulty in distinguishing between the two classes during deep learning. Figure 7 illustrates examples of correctly and incorrectly classified images for Body A and Body B. As shown in the examples, distinguishing features between Body A and Body B is challenging through human visual perception.

### 3.2. Classification Accuracy through Image Preprocessing Using Sharpen and Detail Filters

In diverse scenarios, image preprocessing enhances outcomes. For Experiment 1, the goal was to boost accuracy by applying three image filters—sharpen, detail, and sharpen and detail—across three datasets. The sharpen filter, modeled after the approach of Nascimento et al. [64], demonstrated superior performance improvement compared with the original image. Additionally, findings in studies by Hentschel et al. [65] suggested that the detail filter, which increases sharpness, enhances image quality. To enhance the accuracy of the classification of upper gastrointestinal landmarks, sharpen and detail filters were applied. Both filters were imported and implemented using Python’s scikit-image package [66], resulting in the creation of nine new datasets. The anticipation was that the sharpen filter would excel in highlighting feature points and the detail filter would improve accuracy by expressing points in detail. The combination of both filters in the sharpen and detail filter was hypothesized to yield the highest accuracy. Training was conducted with these nine datasets using five different image sizes, following the same learning environment as in Experiment 1. The results are presented in Table 7, and the reliability of these results was enhanced through the inclusion of precision, recall, and the F1 measure as evaluation indicators.

In this experiment, an evaluation index utilizing a confusion matrix is presented. Evaluation indicators include precision, recall, and F1 measures, along with accuracy. The formula for each indicator is as follows:

(4) $$Accuracy=\frac{TP+TM}{TP+FP+FN+TN}$$

(5) $$Precision=\frac{TP}{TP+FP}$$

(6) $$Recall=\frac{TP}{TP+FN}$$

(7) $$F1\;measure=2\times \frac{Precision\times Recall}{Precision+Recall}$$

#### 3.2.1. Accuracy Comparison between the Datasets

In this comprehensive analysis, the classification accuracy of DenseNet169-based datasets across various factors was assessed. The results indicated that datasets with sharpen and detail filters exhibited the highest classification accuracy. Applying the sharpen filter led to a slight increase in accuracy, whereas the detail filter, when applied alone, showed no significant improvement. However, simultaneous application of both filters resulted in an overall accuracy increase compared with that achieved on the baseline dataset. These outcomes highlight the enhanced accuracy achievable with datasets that feature the sharpen and detail filters.

#### 3.2.2. Classification Accuracy across Different Input Image Sizes

Among the five image sizes tested, 128 × 128 and 320 × 320 demonstrated the highest average accuracy. Notably, the 512 × 512 size of the R sharpen and detail dataset exhibited the highest accuracy improvement—approximately 2.74%, rising from 91.32% in the original R dataset to 94.06%. The second-highest improvement was observed in the 512 × 512 size of the G sharpen and detail dataset, which increased by 1.59% from 91.87% to 93.46% of the original R dataset. While DenseNet169’s average accuracy was primarily notable in the 128 × 128 video size section, applying the sharpen and detail filter resulted in improved classification accuracy even for larger image sizes, contrary to prior experimental results.

#### 3.2.3. Classification Accuracy among Different Classes

**Figure 8:**
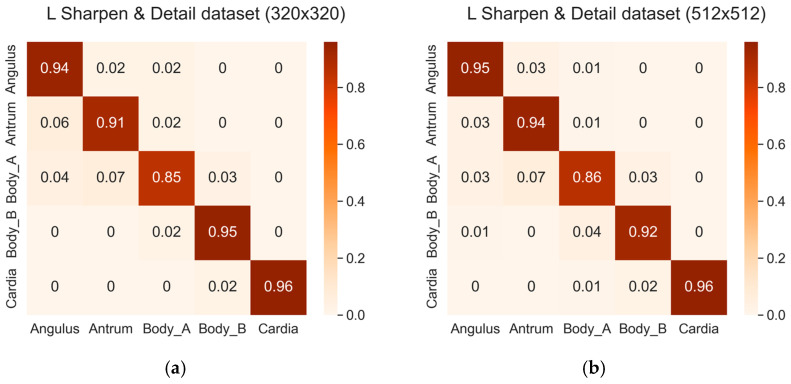

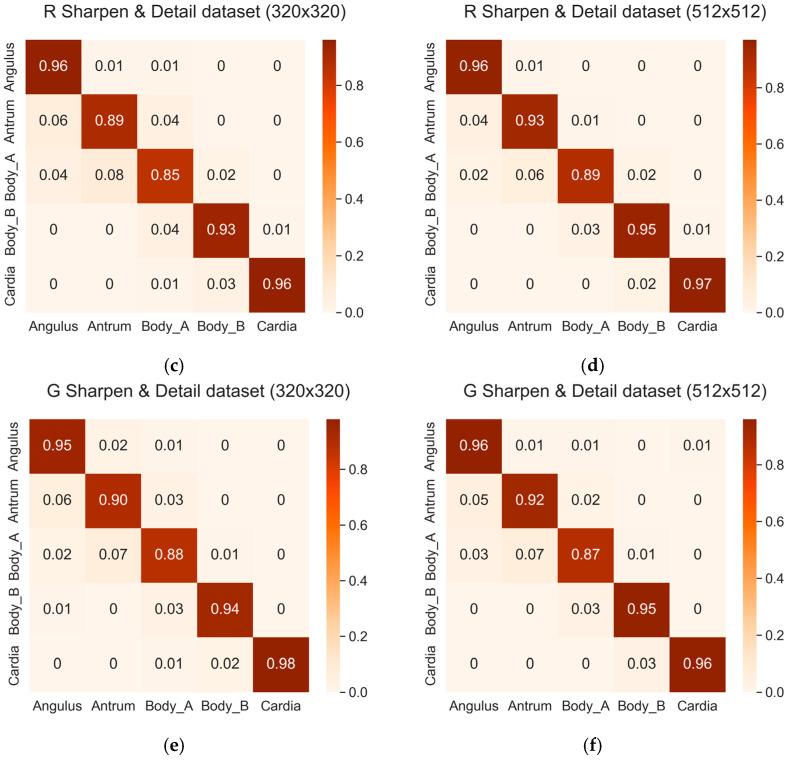
Confusion matrices of the three datasets with the sharpen and detail filter: (**a**) L sharpen and detail dataset (320 × 320); (**b**) Z sharpen and detail dataset (512 × 512); (**c**) R sharpen and detail dataset (320 × 320); (**d**) R sharpen and detail dataset (512 × 512); (**e**) G sharpen and detail dataset (320 × 320); (**f**) G sharpen and detail dataset (512 × 512).

Among the five classes, the highest accuracy was achieved for the Cardia and Fundus class, whereas the lowest accuracy was achieved for the Body A class, consistent with the findings in Section 3.1. However, it was observed that the probability of misclassification for each class decreased with the application of image filters. Figure 8 presents the confusion matrices of the three datasets with the sharpen and detail filter applied. The left matrix corresponds to a size of 320 × 320, and the right matrix corresponds to a size of 512 × 512.

## 4. Discussion

In this experiment, the absence of commercially available upper gastrointestinal landmark datasets was addressed by generating and validating wireless capsule endoscopy (WCE) data based on a developed hypothesis. Initially, the image similarity in the small intestine dataset with real WCE images through color transfer was established, extending the same technique to the upper gastrointestinal tract dataset. While the ideal enhancement would involve using actual upper gastrointestinal landmark WCE images, the absence of a commercially available active capsule endoscope capable of imaging upper gastrointestinal landmarks led us to conduct experiments with datasets resembling the real environment. Obtaining real data for upper gastrointestinal landmarks in the future would significantly enhance the reliability of validating image similarity with our hypothesized dataset. The class-based classification accuracy results for the five landmarks were crucial. Across several experiments, high accuracy was achieved for the Cardia and Fundus classes, with the second-highest accuracy exhibited for the Angulus class. These classes, distinguished by the shape of the stomach folds and the stomach wall contributed to their high accuracy. In contrast, Body A and Body B exhibited the lowest average classification probability among the five classes, indicating unclear differences between images belonging to these two classes. It is hoped that collecting more data and refining the model structure will lead to improved accuracy, which will require further research for a clearer classification of these two classes in the future. And, the DenseNet169 model exhibited notable efficacy in leveraging learned features and facilitating gradient propagation, particularly in the context of increasingly high-resolution medical imaging data. However, its deep architecture and dense connectivity contribute to substantial memory usage, requiring considerable computational resources and time for training. To address these challenges and ensure responsiveness while conserving resources, especially at elevated resolutions, there is a pressing need for refinements and adjustments in the model design. This study also explored the impact of image size on classification accuracy, revealing a decrease in accuracy as image size increased. To enhance overall classification accuracy, sharpen and detail filters were employed among other image filters in the second experiment, resulting in improved accuracy for all image sizes. This finding holds promise for stimulating medical imaging studies involving classification.

## 5. Conclusions

This study established a hypothesis to simulate wireless capsule endoscopy (WCE) images for upper gastrointestinal landmarks by creating 6400 images augmented from 2546 datasets and validated the hypothesis through dataset collection. The Euclidean distance was subsequently applied to assess image similarity. The DenseNet169 model confirmed the highly similar accuracy of the three datasets to the WCE images. Furthermore, training DenseNet169 with a preprocessed dataset incorporating image filters demonstrated improved accuracy compared with the previous experiment.

In the evolving market for endoscopy equipment, our research focused on identifying landmarks captured using future active capsule endoscopes and enhancing the accuracy of the classification of capsule endoscope images through image preprocessing, utilizing color transfer techniques similar to those employed for wired endoscope images. While current wired endoscopes offer high image quality and clear vision, capsule endoscope imaging still requires further enhancements in image quality. Our research findings indicate that, through image preprocessing, deep learning models trained on capsule endoscope images can achieve accuracy comparable to those trained using wired endoscope images, facilitated by sharpening and detail filtering. The medical significance lies in the fact that image preprocessing allows capsule endoscopy images to be used produce results similar to those of wired endoscopy even under conditions with lower image quality. It is anticipated that these advancements will enable more precise diagnostic assistance in gastroscopy through images captured with future capsule endoscopy equipment for the upper gastrointestinal tract.

## Figures and Tables

**Table 1:** Studies on landmarks in wireless capsule endoscopy by year.

Autor	Year	Landmark Regions	CNN	Applications	Accuracy
Rahman et al. [29]	2016	Upper stomach	-	MACE	88–100%
Chen et al. [30]	2017	Esophagus, stomach, Small intestine, colon	N-CNN, O-CNN, etc.	WCE, HMM	78–97%
Jang et al. [31]	2020	Stomach, small intestine, Large intestine	Purposed CNN	WCE, GTA	95%, 71%
Adewole et al. [14]	2021	Esophagus, stomach, Small intestine, colon	VGG19, GoogleNet, ResNet50, AlexNet	VCE, Grad-CAM	85.40% 99.10%
Wang et al. [32]	2022	Small intestine	VGG16, VGG19, DenseNet121, DenseNet201, AGDN, InceptionV3, etc.	WCE, CNN feature extraction module	94.83% 85.99%
Alam et al. [33]	2022	Small intestine	Rat-CapsNet	WCE, VAM	98.51%, 95.65%
Pascual et al. [34]	2022	Small intestine, large intestine	ResNet50	WCE, SSL	95.00% 92.77%
Athanasiou et al. [35]	2023	Esophagus, stomach, Small intestine, large intestine	Purposed 3 CNN	WCE, CAD	95.56%
Laiz et al. [36]	2023	Small intestine, large intestine	ResNet50	WCE, CMT Time Block	91.36% 94.58% 99.09%
Vaghela et al. [37]	2023	Small intestine	DCAN-DenseNet	WCE, SR	94.86% 93.78%

**Table 2:** Accuracy comparison of five models on the original dataset.

Model	Accuracy
ResNet50	92.03%
ResNet50V2	90.58%
InceptionV3	89.83%
DenseNet121	92.26%
DenseNet169	93.28%
DenseNet201	91.17%

**Table 3:** Distribution of images in the original dataset.

Class	Images
Angulus	493
Antrum	901
Body A	533
Body B	392
Cardia and Fundus	207

**Table 4:** Euclidean distance measurements for color-transferred Kvasir dataset and Kvasir-capsule dataset.

Color Transfer Kvasir Class	Kvasir-Capsule Image (1)	Kvasir-Capsule Image (2)
Normal cecum	0.90–1.18	0.89–1.19
Normal pylorus	0.78–1.21	0.82–1.24
Normal z line	0.77–1.24	0.81–1.21

**Table 5:** Euclidean distance measurement results for the target image and three color-transferred datasets, indicating the measured values for each class across 1280 images.

Dataset	Angulus	Antrum	Body A	Body B	Cardia and Fundus
L dataset	0.92–1.20	0.90–1.17	0.93–1.20	0.93–1.20	0.93–1.20
R dataset	0.93–1.21	0.90–1.20	0.87–1.19	0.90–1.18	0.93–1.20
G dataset	0.78–1.16	0.76–1.10	0.76–1.13	0.84–1.15	0.82–1.19

**Table 6:** Comparison of results across five input sizes for L, R, and G datasets.

Dataset	128 × 128	256 × 256	320 × 320	384 × 384	512 × 512
L dataset	93.20%	92.89%	92.26%	92.57%	92.18%
R dataset	92.65%	91.40%	92.34%	92.73%	91.87%
G dataset	93.20%	92.73%	92.18%	91.71%	91.32%

**Table 7:** Comparative results of 9 datasets with applied sharpen and detail filters for 5 input sizes. (Acc: accuracy, Pre: precision, Rec: recall, F1: F1 measure).

Dataset	128 × 128				256 × 256				320 × 320				384 × 384				512 × 512			
	Acc	Pre	Rec	F1	Acc	Pre	Rec	F1	Acc	Pre	Rec	F1	Acc	Pre	Rec	F1	Acc	Pre	Rec	F1
L Sharpen	93.04	93.38	92.56	92.96	92.57	93.00	92.42	92.70	92.73	93.29	92.42	92.70	93.04	93.60	92.65	93.12	92.81	93.15	92.50	92.82
L Detail	92.18	92.66	91.79	92.22	93.12	93.43	92.73	93.07	92.73	93.37	92.42	92.89	92.50	93.04	92.26	92.64	92.65	93.50	92.18	92.83
L Sharpen & Detail	93.20	93.47	92.96	93.21	93.34	93.64	93.20	93.69	93.35	93.92	93.04	93.47	93.28	93.75	92.50	93.12	92.96	93.73	92.26	92.98
R Sharpen	93.28	93.63	93.04	93.33	92.57	92.99	92.34	92.66	93.12	93.70	92.96	93.32	92.57	93.13	92.18	92.65	92.50	93.16	91.56	92.35
R Detail	92.65	92.99	92.26	92.62	91.32	91.67	91.17	91.41	92.34	93.20	92.10	92.64	92.73	93.29	92.42	92.85	91.40	93.29	92.42	92.85
R Sharpen & Detail	93.65	93.86	93.28	93.56	93.04	93.30	92.57	92.93	93.20	93.69	92.81	93.24	93.20	93.69	92.89	93.28	94.06	94.55	93.67	94.10
G Sharpen	92.96	93.26	93.23	93.24	92.65	93.28	92.26	92.76	92.34	92.68	92.10	92.38	92.26	93.22	92.50	92.85	93.20	93.40	92.89	93.14
G Detail	92.73	93.08	92.50	92.78	92.26	92.65	91.64	92.14	92.50	92.84	92.18	92.50	92.89	93.42	92.10	92.75	91.40	92.84	92.18	92.50
G Sharpen & Detail	93.43	93.66	93.43	93.54	92.89	93.24	92.73	92.98	93.04	93.31	92.73	93.01	93.04	93.45	92.57	93.00	93.46	93.68	92.65	93.16

## Data Availability

The data presented in this study are available on request from the corresponding author (accurately indicate status).

## References

[1] Siddhi S., Dhar A., Sebastian S. (2021). Best practices in environmental advocacy and research in endoscopy. Tech. Innov. Gastrointest. Endosc..

[2] Xiao Z., Lu J., Wang X., Li N. (2023). WCE-DCGAN: A data augmentation method based on wireless capsule endoscopy images for gastrointestinal disease detection. IET Image Process..

[3] Soffer S., Klang E., Shimon O., Nachmias N., Eliakim R., Benhorin S., Kopylov U., Barash Y. (2020). Deep learning for wireless capsule endoscopy: A systematic review and meta-analysis. Gastrointest. Endosc..

[4] Li B., Meng M.Q.H. (2012). Tumor recognition in wireless capsule endoscopy images using textural features and SVM-based feature selection. IEEE Trans. Inf. Technol. Biomed..

[5] Lonescu A.G., Glodeanu A.D., Lonescu M., Zaharie S.L., Ciurea A.M., Golli A.L., Mavritsakis N., Popa D.L., Vere C.C. (2022). Clinical impact of wireless capsule endoscopy for small bowel investigation. Exp. Ther. Med..

[6] Saito H., Aoki T., Mmath K.A., Kato Y., Tsuboi A., Yamada A., Fujishiro M., Oka S., Ishihara S., Matsuda T. (2020). Automatic detection and classification of protruding lesions in wireless capsule endoscopy images based on a deep convolutional neural network. Gastrointest. Endosc..

[7] Zhang Y., Zhang Y., Huang X. (2021). Development and application of magnetically controlled capsule endoscopy in detecting gastric lesions. Gastroenterol. Res. Pract..

[8] Jiang X., Pan J., Li Z.S., Liao Z. (2019). Standardized examination procedure of magnetically controlled capsule endoscopy. VideoGIE.

[9] Hoang M.C., Nguyen K.T., Le V.H., Kim J., Choi E., Kang B., Park J.O., Kim C.S. (2019). Independent electromagnetic field control for practical approach to actively locomotive wireless capsule endoscope. IEEE Trans. Syst. Man Cybern. Syst..

[10] Zhang Y., Qu L., Hao J., Pan Y., Huang X. (2022). In vitro and in vivo evaluation of a novel wired transmission magnetically controlled capsule endoscopy system for upper gastrointestinal examination. Surg. Endosc..

[11] Kim J.H., Nam S.J. (2021). Capsule endoscopy for gastric evaluation. Diagnostics.

[12] Serrat J.A.A., Córdova H., Moreira L., Pocurull A., Ureña R., Delgadoguillena P.G., Garcésdurán R., Sendino O., Garcíarodríguez A., Gonzálezsuárez B. (2020). Evaluation of long-term adherence to oesophagogastroduodenoscopy quality indicators. Gastroenterol. Hepatol. (Engl. Ed.).

[13] Tran T.H., Nguyen P.T., Tran D.H., Manh X.H., Vu D.H., Ho N.K., Do K.L., Nguyen V.T., Nguyen L.T., Dao V.H. (2021). Classification of anatomical landmarks from upper gastrointestinal endoscopic images. Proceedings of the 2021 8th NAFOSTED Conference on Information and Computer Science (NICS).

[14] Adewole S., Yeghyayan M., Hyatt D., Ehsan L., Jablonski J., Copland A., Syed S., Brown D. (2021). Deep learning methods for anatomical landmark detection in video capsule endoscopy images. Proceedings of the Future Technologies Conference (FTC) 2020.

[15] Xu Z., Tao Y., Wenfang Z., Ne L., Zhengxing H., Jiquan L., Wiling H., Huilong D., Jianmin S. (2019). Upper gastrointestinal anatomy detection with multi-task convolutional neural networks. Healthc. Technol. Lett..

[16] Takiyama H., Ozawa T., Ishihara S., Fujishiro M., Shichijo S., Nomura S., Miura M., Tada T. (2018). Automatic anatomical classification of esophagogastroduodenoscopy images using deep convolutional neural networks. Sci. Rep..

[17] Cogan T., Cogan M., Tamil L. (2019). MAPGI: Accurate identification of anatomical landmarks and diseased tissue in gastrointestinal tract using deep learning. Comput. Biol. Med..

[18] Jha D., Ali S., Hicks S., Thambawita V., Borgli H., Smedsrud P.H., de Lange T., Pogorelov K., Wang X., Harzig P. (2021). A comprehensive analysis of classification methods in gastrointestinal endoscopy imaging. Med. Image Anal..

[19] Smedsrud P.H., Thambawita V., Hicks S.A., Gjestang H., Nedrejord O.O., Næss E., Borgli H., Jha D., Berstad T.J.B., Eskeland S.L. (2021). Kvasir-Capsule, a video capsule endoscopy dataset. Sci. Data.

[20] Jha D., Smedsrud P.H., Riegler M.A., Halvorsen P., Lange T., Johansen D., Johansen H. (2020). Kvasir-seg: A segmented polyp dataset. MultiMedia Modeling: 26th International Conference, MMM 2020, Daejeon, South Korea, January 5–8 2020, Proceedings, Part II 26.

[21] Pogorelov K., Randel K.R., Griwodz C., Eskeland S.L., Lange T., Johansen D., Spampinato C., Dangnguyen D.T., Lux M., Schmidt P.T. Kvasir: A multi-class image dataset for computer aided gastrointestinal disease detection. Proceedings of the Proceedings of the 8th ACM on Multimedia Systems Conference.

[22] Crum W.R., Camara O., Hill D.L.G. (2006). Generalized overlap measures for evaluation and validation in medical image analysis. IEEE Trans. Med. Imaging.

[23] Tian H., Li Y., Pian W., Kaboré A.K., Liu K., Habib A., Klein J., Bissyandé T.F. (2022). Predicting patch correctness based on the similarity of failing test cases. ACM Trans. Softw. Eng. Methodol. (TOSEM).

[24] Kandel I., Castelli M. (2021). Transfer learning with convolutional neural networks for diabetic retinopathy image classification: A review. Appl. Sci..

[25] Zhang W., Zhong J., Yang S., Gao Z., Hu J., Chen Y., Yi Z. (2019). Automated identification and grading system of diabetic retinopathy using deep neural networks. Knowl.-Based Syst..

[26] Krizhevsky A., Sutskever I., Hinton G.E. (2012). Imagenet classification with deep convolutional neural networks. Adv. Neural Inf. Process. Syst..

[27] Kaiming H., Zhang X., Ren S., Sun J. Deep residual learning for image recognition. Proceedings of the IEEE Conference on Computer Vision and Pattern Recognition.

[28] Szegedy C., Liu W., Jia Y., Sermanet P., Reed S., Anguelov D., Erhan D., Vanhoucke V., Rabinovich A. Going deeper with convolutions. Proceedings of the IEEE Conference on Computer Vision and Pattern Recognition.

[29] Rahman M.T., Dola A. (2021). Automated grading of diabetic retinopathy using densenet-169 architecture. Proceedings of the 2021 5th International Conference on Electrical Information and Communication Technology (EICT).

[30] Chen H., Wu X., Tao G., Peng Q. (2017). Automatic content understanding with cascaded spatial–temporal deep framework for capsule endoscopy videos. Neurocomputing.

[31] Jang H.W., Lim C.N., Park Y.S., Lee G.J., Lee J.W. (2020). Estimating gastrointestinal transition location using CNN-based gastrointestinal landmark classifier. KIPS Trans. Softw. Data Eng..

[32] Wang W., Yang X., Li X., Tang J. (2022). Convolutional-capsule network for gastrointestinal endoscopy image classification. Int. J. Intell. Syst..

[33] Alam J., Rashid R.B., Fattah S.A., Saquib M. (2022). RAt-CapsNet: A Deep Learning Network Utilizing Attention and Regional Information for Abnormality Detection in Wireless Capsule Endoscopy. IEEE J. Transl. Eng. Health Med..

[34] Pascual G., Laiz P., García A., Wenzek H., Vitrià J., Seguí S. (2022). Time-based self-supervised learning for Wireless Capsule Endoscopy. Comput. Biol. Med..

[35] Athanasiou S.A., Sergaki E.S., Polydorou A.A., Stavrakakis G.S., Afentakis N.M., Vardiambasis I.O., Zervakis M.E. (2023). Revealing the Boundaries of Selected Gastro-Intestinal (GI) Organs by Implementing CNNs in Endoscopic Capsule Images. Diagnostics.

[36] Laiz P., Vitrià J., Gilabert P., Wenzek H., Malagelada C., Watson A.J.M., Seguí S. (2023). Anatomical landmarks localization for capsule endoscopy studies. Comput. Med. Imaging Graph..

[37] Vaghela H., Sarvaiya A., Premlani P., Agarwal A., Upla K., Raja K., Pedersen M. (2023). DCAN: DenseNet with Channel Attention Network for Super-resolution of Wireless Capsule Endoscopy. Proceedings of the 2023 11th European Workshop on Visual Information Processing.

[38] Cai Q., Lis X., Guo Z. (2018). Identifying architectural distortion in mammogram images via a se-densenet model and twice transfer learning. Proceedings of the 2018 11th International Congress on Image and Signal Processing, BioMedical Engineering and Informatics (CISP-BMEI).

[39] Vulli A., Srinivasu P.N., Sashank M.S.K., Shafi J., Choi J., Ijaz M.F. (2022). Fine-tuned DenseNet-169 for breast cancer metastasis prediction using FastAI and 1-cycle policy. Sensors.

[40] Fekriershad S., Alimari M.J., Hamad M.H., Alsaffar M.F., Hassan F.G., Hadi M.E., Mahdi K.S. (2022). Cell phenotype classification based on joint of texture information and multilayer feature extraction in DenseNet. Comput. Intell. Neurosci..

[41] Abbas Q., Qureshi I., Ibrahim M.E.A. (2021). An Automatic Detection and Classification System of Five Stages for Hypertensive Retinopathy Using Semantic and Instance Segmentation in DenseNet Architecture. Sensors.

[42] Farag M.M., Fouad M., Abdelhamid A.T. (2022). Automatic severity classification of diabetic retinopathy based on densenet and convolutional block attention module. IEEE Access.

[43] Varma N.M., Choudhary A. (2019). Evaluation of Distance Measures in Content Based Image Retrieval. Proceedings of the 2019 3rd International conference on Electronics, Communication and Aerospace Technology (ICECA).

[44] Korenius T., Laurikkala J., Juhola M. (2007). On principal component analysis, cosine and Euclidean measures in information retrieval. Inf. Sci..

[45] Srikaewsiew T., Khianchainat K., Tharatipyakul A., Pongnumkul S., Kanjanawattana S. (2022). A Comparison of the Instructor-Trainee Dance Dataset Using Cosine similarity, Euclidean distance, and Angular difference. Proceedings of the 2022 26th International Computer Science and Engineering Conference (ICSEC).

[46] Zenggang X., Zhiwen T., Xiaowen C., Xuemin Z., Kaibin Z., Conghuan Y. (2021). Research on image retrieval algorithm based on combination of color and shape features. J. Signal Process. Syst..

[47] Heidari H., Chalechale A., Mohammadabadi A.A. (2013). Parallel implementation of color based image retrieval using CUDA on the GPU. Int. J. Inf. Technol. Comput. Sci. (IJITCS).

[48] Wang L., Zhang Y., Feng J. (2005). On the Euclidean distance of images. IEEE Trans. Pattern Anal. Mach. Intell..

[49] Xia P., Zhang L., Li F. (2015). Learning similarity with cosine similarity ensemble. Inf. Sci..

[50] Zhang D., Lu G. (2003). Evaluation of similarity measurement for image retrieval. Proceedings of the International Conference on Neural Networks and Signal Processing 2003. Proceedings of the 2003.

[51] Ferreira J.R., Oliveira M.C., Freitas A.L. (2014). Performance evaluation of medical image similarity analysis in a heterogeneous architecture. Proceedings of the 2014 IEEE 27th International Symposium on Computer-Based Medical Systems.

[52] Garcia N., Vogiatzis G. (2019). Learning non-metric visual similarity for image retrieval. Image Vis. Comput..

[53] Reinhard E., Adhikhmin M., Gooch B., Shirley P. (2001). Color transfer between images. IEEE Comput. Graph. Appl..

[54] Yin L., Jia J., Morrissey J. (2004). Towards race-related face identification: Research on skin color transfer. Proceedings of the Sixth IEEE International Conference on Automatic Face and Gesture Recognition, 2004. Proceedings.

[55] Mohapatra S., Pati G.K., Mishra M., Swarnkar T. (2023). Gastrointestinal abnormality detection and classification using empirical wavelet transform and deep convolutional neural network from endoscopic images. Ain Shams Eng. J..

[56] Liao Z., Duan X.D., Xin L., Bo L.M., Wang X.H., Xiao G.H., Hu L.H., Zhuang S.L., Li Z.S. (2012). Feasibility and safety of magnetic-controlled capsule endoscopy system in examination of human stomach: A pilot study in healthy volunteers. J. Interv. Gastroenterol..

[57] Rahman I., Pioche M., Shim C.S., Lee S.P., Sung I.K., Saurin J.C.S., Patel P. (2016). Magnetic-assisted capsule endoscopy in the upper GI tract by using a novel navigation system (with video). Gastrointest. Endosc..

[58] Khan A., Malik H. Gastrointestinal Bleeding WCE images Dataset 2023. https://data.mendeley.com/datasets/8pbbjf274w/1.

[59] Jain S., Seal A., Ojha A., Yazidi A., Bures J., Tacheci I., Krejcar O. (2021). A deep CNN model for anomaly detection and localization in wireless capsule endoscopy images. Comput. Biol. Med..

[60] Iqbal I., Walayat K., Kakar M.U., Ma J. (2022). Automated identification of human gastrointestinal tract abnormalities based on deep convolutional neural network with endoscopic images. Intell. Syst. Appl..

[61] Handa P., Goel N., Indu S. (2022). Automatic intestinal content classification using transfer learning architectures. Proceedings of the 2022 IEEE International Conference on Electronics, Computing and Communication Technologies (CONECCT).

[62] Sushma B., Aparna P. (2022). Recent developments in wireless capsule endoscopy imaging: Compression and summarization techniques. Comput. Biol. Med..

[63] Panetta K., Bao L., Agaian S. (2016). Novel multi-color transfer algorithms and quality measure. IEEE Trans. Consum. Electron..

[64] Nascimento H.A.R., Ramos A.C.A., Neves F.S., Deazevedovaz S.L., Freitas D.Q. (2015). The ‘Sharpen’ filter improves the radiographic detection of vertical root fractures. Int. Endod. J..

[65] Hentschel C., Lahei D. (2001). Effective peaking filter and its implementation on a programmable architecture. IEEE Trans. Consum. Electron..

[66] Walt S.V.D., Schönberger J.L., NunezIglesias J., Boulogne F., Warner J.D., Yager N., Gouillart E., Yu T. (2014). scikit-image: Image processing in Python. PeerJ.

